# Effects of magnesium supplementation on improving hyperglycemia, hypercholesterolemia, and hypertension in type 2 diabetes: A pooled analysis of 24 randomized controlled trials

**DOI:** 10.3389/fnut.2022.1020327

**Published:** 2023-01-18

**Authors:** Lianbin Xu, Xiuli Li, Xinhui Wang, Mingqing Xu

**Affiliations:** ^1^College of Animal Science and Technology, Qingdao Agricultural University, Qingdao, China; ^2^College of Veterinary Medicine, Qingdao Agricultural University, Qingdao, China; ^3^School of Public Health, Zhejiang University School of Medicine, Hangzhou, China; ^4^Key Laboratory for the Genetics of Developmental and Neuropsychiatric Disorders (Ministry of Education), Bio-X Institutes, Shanghai Jiao Tong University, Shanghai, China; ^5^Center for Biomedical Informatics, Harvard Medical School, Boston, MA, United States

**Keywords:** blood pressure, glycemic control, serum lipids, magnesium supplementation, optimal details, type 2 diabetes

## Abstract

**Background:**

Previous studies have demonstrated that diabetes is often accompanied with lower magnesium status. However, practical details regarding the influences of magnesium intervention on hyperglycemia, hypercholesterolemia, and hypertension in type 2 diabetes (T2D) need to be further investigated.

**Methods:**

Web of Science, ScienceDirect, and PubMed were searched for relevant literatures published through April 30, 2022, and high-quality data were pooled to evaluate the effects of magnesium supplementation on glycemic, circulating lipids, and blood pressure control in T2D, and to explore the associated practical details.

**Results:**

Pooled analyses of 24 randomized controlled trials with 1,325 T2D individuals revealed that subjects who received magnesium supplementation had statistically significant reductions in fasting plasma glucose, glycated hemoglobin, systolic blood pressure and diastolic blood pressure, with WMD values of –0.20 mM (95% CI: –0.30, –0.09), –0.22% (95% CI: –0.41, –0.03), –7.69 mmHg (95% CI: –11.71, –3.66) and –2.71 mmHg (95% CI: –4.02, –1.40), respectively. Detailed subgroup analyses demonstrated that health status of participants including age, body mass index, country, duration of disease, baseline magnesium level and baseline glycemic control condition as well as magnesium formulation, dosage and duration of intervention influenced the effects of magnesium addition. Dose-effect analysis showed that 279 mg/d for 116 d, 429 mg/d for 88 d and 300 mg/d for 120 d are the average optimal dosages and durations for improving glycemic, circulating lipids, and blood pressure controls, respectively.

**Conclusion:**

Our findings provide clinically relevant information on the adjuvant therapy of magnesium for improving hyperglycemia, hypercholesterolemia, and hypertension in T2D.

## Introduction

The diabetes prevalence is predicted to be 10.9% by 2045 worldwide, which has negative effects on the well-being of individuals (1). Type 2 diabetes (T2D) is a common metabolic disorder usually accompanied with β cell impairment, insulin resistance and hyperglycemia (2), leading to a diminished glucose control. Previous work established that T2D individuals have significant positive relations to formations of hypertension and hypercholesterolemia (3), which are the main risk factors related to cardiovascular diseases resulting in high mortality worldwide (4). Therefore, populations with T2D appear to own common syndromes of increased plasma glucose, reduced insulin sensitivity, hypertension and dyslipidemia simultaneously (5), highlighting the importance to find ways to treat these complications concurrently.

Magnesium plays a key role in many metabolisms as a cofactor of enzymatic pathways (6). Previous work showed that hypomagnesemia was reported in about 30% of diabetic patients (7). Accumulating evidence demonstrated that higher magnesium intake improved insulin release and sensibility (8, 9), dyslipidemia (10), and dysfunction of endothelial cells (11), and reduced thrombotic tendency (12) and vascular contractility (13). Therefore, clinical magnesium supplementation may be a strategy to improve the outcomes of T2D cases.

Several systematic reviews (14, 15) carried out on randomized controlled trials (RCT) were performed to examine the beneficial influences of magnesium intervention on development of T2D, but the results were less conclusive. Furthermore, meta-analysis that simultaneous investigated the influences of magnesium intervention on hyperglycemia, hypertension and hyperlipidemia in T2D is relatively limited. Thus, the effects of magnesium addition improving the parameters related to the complications of T2D, as well as the associated practical issues, require additional investigation.

We hypothesized that the dosage effects of magnesium addition on clinical outcomes of T2D depend on the disease status and mode of intervention. Therefore, in this meta-analytic study, we collected RCT data of updated studies to investigate the efficacy of magnesium supplementation on glycemic, plasma lipids and blood pressure controls in T2D, and to explore the optimal details associated with this strategy based on the patient’s health status and mode of intervention through subgroup and dose-effect analyses.

## Methods

### Data collection

**FIGURE 1:**
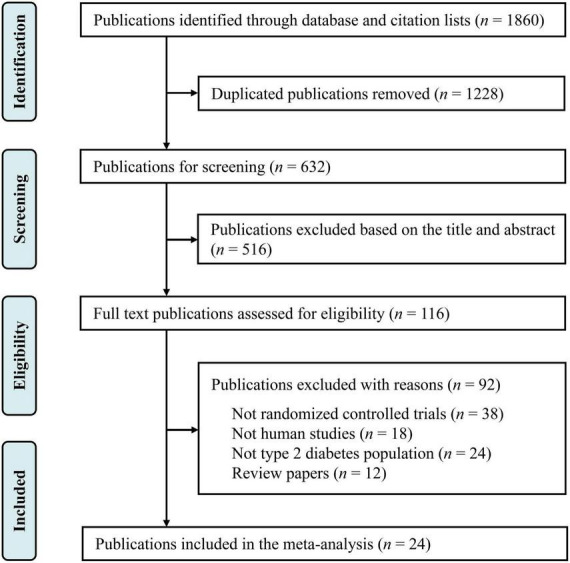
Flowchart of the study design.

This study was registered in the International Prospective Register of Systematic Reviews (PROSPERO) database under CRD42022324969, and we followed the Preferred Reporting Items for Systematic Reviews and Meta-Analyses (PRISMA) instructions (16). Relevant published studies were searched in Web of Science, PubMed and ScienceDirect published through April 30, 2022, with the following keywords: (“magnesium supplementation” OR “magnesium intervention” OR “magnesium”) AND (“diabetes” OR “type 2 diabetes” OR “non-insulin-dependent diabetics”) AND [“glucose” OR “fasting plasma glucose (FPG) OR “glycemia” OR “glycemic control” OR “insulin” OR “glycated hemoglobin (HbA1c)” OR “homeostasis model assessment-insulin resistance (HOMA-IR)”] AND [“lipids” OR “total cholesterol (TC)” OR “total triglyceride (TG)” OR “low density lipoprotein cholesterol (LDL-C)” OR “high density lipoprotein cholesterol (HDL-C)”] AND [“blood pressure” OR “systolic blood pressure (SBP)” OR “diastolic blood pressure (DBP)”]. There were no limitations in the language of studies. Additional publications were further collected through reviewing the references of selected relevant publications. The selection process is summarized in Figure 1.

### Criteria on included study

Studies met the following criteria were included in this meta-analysis: (i) being a parallel or cross-over design in RCT, (ii) exploring the influences of magnesium addition in T2D patients, (iii) reporting data on one or more of the following items: FPG, insulin, HDL-C, LDL-C, TC and TG concentrations along with HbA1c, HOMA-IR, SBP and DBP values; (iv) reporting the information on above items at baseline and at the end of follow-up. Exclusion criteria were (i) the study was not a RCT, (ii) the study had no control group, (iii) the study had a case-control, cross-sectional or cohort design, and (iv) the study didn’t report information related to baseline or follow-up parameters; (v) the study was a methodologic report, review, comment or abstract.

### Data extraction

Detailed information was collected from the eligible studies, containing the first author’s name, study region, sample size, participant age and body mass index (BMI), duration of diabetes, dosage and duration of magnesium intervention, and plasma concentrations of magnesium, glucose, insulin, HDL-C, LDL-C, TC and TG as well as HbA1c, HOMA-IR, SBP and DBP values. The units of FBS, insulin and HbA1c were converted to mM, mU/L and percentage, respectively. Besides, the HDL-C, LDL-C, TC and TG concentrations were all collated in mg/dL.

### Quality assessment

The methodologic quality of the RCTs were assessed by Jadad scale (17). Each publication was assigned a score from 0 (“poor”) to 5 (“good”) according to the criteria: (i) does the article have a randomized design? (ii) does the article have a double-blind design? (iii) does the article report withdrawals and dropouts? (iv) does the article describe the randomization procedures and they are appropriate? (v) does the study report appropriate blinding techniques? Each “yes” or “no” response will get 1 or 0 points, respectively (Supplementary Table 1).

### Quantitative data synthesis

We used Stata version 14 to perform the statistical analyses. The differences of mean and SD between baseline and endpoint were calculated according to the formula: changes of mean = (measure at endpoint) – (measure at baseline); SD = squareroot [(SD_baseline_)^2^ + (SD_endpoint_)^2^ – (2R × SD_baseline_ × SD_endpoint_)], assuming a correlation coefficient (R) = 0.5. The weighted mean difference (WMD) for continuous outcomes were computed between the magnesium and control groups using a random-effect model. Between-study heterogeneity was assessed using the *I*^2^ statistic, with 0–25%, 25.1–75%, and 75.1–100% representing a low, moderate, or high degree of heterogeneity, respectively (18). Publication bias was measured by the contour-enhanced funnel plots and Egger’s linear regression test, with threshold of significance at *P* < 0.05. The effects of individual studies on the pooled meta-analytic results were determined with the sensitivity analysis (19).

### Subgroup and dose-effect analyses

Subgroup analyses were made according to the participant’s age and BMI, country, duration of disease, baseline magnesium level and baseline glycemic control condition as well as magnesium formulation, dosage and duration of intervention. Meta-regression analyses were used to compare the subgroup differences. In addition, dose-effect model was used to find the optimal dosage and duration of magnesium intervention with the R 4.2.0 software (The R Foundation Conference Committee, USA).

## Results

### Study characteristics

Our primary search identified 1,860 publications in Web of Science, PubMed and ScienceDirect (Figure 1). After removing the duplicate literatures, 116 articles were screened in detail. Among these, 92 records were removed, including 38 publications that were not RCTs, 24 publications that were not conducted in T2D populations, 18 animal trials, and 12 review papers. Finally, 24 publications were included in the final meta-analysis (Table 1).

**TABLE 1:** Characteristics of included studies on magnesium intervention in type 2 diabetes mellitus^a^.

Reference	Country	Intervation type	Sample size	Age, years	BMI, kg/m^2^	Duration of diabetes, years	Elemental magnesium dose, mg/d	Intervation duration, d	Jadad score
Paolisso et al. (20)	Italy	Magnesium pidolate	8	72.2 ± 5.7		11.5 ± 3.1	171	28	3
		Placebo	8	72.2 ± 5.7		11.5 ± 3.1			
Paolisso et al. (21)	Italy	Magnesium pidolate	8	67.6 ± 4.8	30.5 ± 2.1	8.5 ± 3.3	256	28	4
		Placebo	8	67.6 ± 4.8	30.5 ± 2.1	8.5 ± 3.3			
Corica et al. (22)	Italy	Magnesium pidolate	26	63.0 ± 5.0	24.8 ± 0.7	10.7 ± 3.0	394	30	3
		Placebo	17	61.0 ± 3.0	24.4 ± 0.4	9.9 ± 5.0			
Gullestad et al. (23)	Norway	Magnesium-lactate-citrate	25		25.4 ± 3.7	9.8 ± 8.6	365	120	4
		Placebo	29		25.3 ± 4.1	10.1 ± 9.7			
Paolisso et al. (24)	Italy	Magnesium pidolate	9	73.0 ± 7.5	25.8 ± 0.9	7.9 ± 3.6	384	28	4
		Placebo	9	73.0 ± 7.5	25.8 ± 0.9	7.9 ± 3.6			
Purvis et al. (25)	USA	Magnesium chloride	14	53.8 ± 12.8	32.2 ± 7.1		384	42	5
		Placebo	14	53.8 ± 12.8	32.2 ± 7.1				
Eibl et al. (41)	Austria	Magnesium citrate	18	63.0 ± 8.0	27.5 ± 3.2	7.6 ± 6.9	729	90	4
		Placebo	20	54.0 ± 1.5	29.3 ± 5.0	6.1 ± 5.2			
Eriksson and Kohvakka (26)	Finland	Magnesium	27	61.0 ± 10.4	28.9 ± 4.2	10.0 ± 5.2	600	90	4
		Ascorbic acid	27	61.0 ± 10.4	28.9 ± 4.2	10.0 ± 5.2			
de Lordes Lima et al. (7)	Brazil	Magnesium oxide	35	55.4 ± 10.2	25.3 ± 8.0	7.2 ± 4.9	503	30	5
		Placebo	54	55.5 ± 8.3	25.5 ± 6.5	7.3 ± 5.4			
de Lordes Lima et al. (7)	Brazil	Magnesium oxide	39	51.2 ± 11.0	25.5 ± 6.5	7.1 ± 5.5	1006	30	5
		Placebo	54	55.5 ± 8.3	25.5 ± 6.5	7.3 ± 5.4			
de Valk et al. (27)	The Netherlands	Magnesium-aspartate-HCl	25	63.0 ± 8.2	28.7 ± 5.4	16.1 ± 8.1	365	90	3
		Placebo	25	62.0 ± 7.3	27.1 ± 4.5	15.1 ± 7.6			
Rodríguez-Morán and Guerrero-Romero (28)	Mexico	Magnesium chloride	32	59.7 ± 8.3	27.6 ± 9.1	8.8 ± 4.9	450	112	5
		Placebo	31	54.1 ± 9.6	28.6 ± 4.2	9.4 ± 5.5			
Barragán-Rodríguez et al. (29)	Mexico	Magnesium chloride	12	69.0 ± 5.9		11.8 ± 7.9	450	84	3
		Imipremine	9	66.4 ± 6.1		8.6 ± 5.7			
Guerrero-Romero and Rodríguez-Morán (30)	Mexico	Magnesium chloride	40	58.9 ± 8.5	29.9 ± 5.2	10.4 ± 6.3	450	120	5
		Placebo	39	60.5 ± 9.4	29.0 ± 5.1	10.5 ± 6.0			
Barbagallo et al. (31)	Italy	Magnesium pidolate	30	71.0 ± 4.9			368	30	3
		Placebo	30	71.2 ± 4.6					
Bhardwaj et al. (32)	India	Magnesium chloride	30				300	28, 56 and 112	3
		Placebo	30						
Navarrete-Cortes et al. (33)	Mexico	Magnesium lactate	56	52.8 ± 8.4	30.5 ± 5.7		360	90	5
		Placebo	56	52.8 ± 8.4	30.5 ± 5.8				
Solati et al. (34)	Iran	Magnesium sulphate	25	46.8 ± 9.0	26.2 ± 2.9	4.1 ± 4.2	300	90	5
		Placebo	22	50.2 ± 6.9	26.9 ± 5.2	5.4 ± 4.0			
Singh et al. (35)	India	Magnesium chloride	60				300	28, 56 and 112	3
		Placebo	60						
ELDerawi et al. (36)	Gaza	Magnesium	20	51.2 ± 7.0	29.0 ± 5.1		250	90	3
		Placebo	20	51.6 ± 8.3	30.0 ± 4.6				
Razzaghi et al. (37)	Iran	Magnesium oxide	35	60.1 ± 11.1	28.2 ± 5.2		250	84	5
		Placebo	35	59.0 ± 10.1	26.2 ± 4.1				
Rashvand et al. (38)	Iran	Magnesium oxide	18	49.9 ± 7.8	29.7 ± 3.2	6.5 ± 3.4	500	60	5
		Placebo	19	48.2 ± 14.2	29.3 ± 3.7	5.8 ± 3.1			
Talari et al. (39)	Iran	Magnesium oxide	27	58.8 ± 10.1	27.2 ± 5.6	4.0 ± 1.0	250	168	5
		Placebo	27	61.8 ± 10.2	26.1 ± 4.5	3.8 ± 1.0			
Rashvand et al. (42)	Iran	Magnesium oxide	18	49.9 ± 7.8	29.7 ± 3.2	6.5 ± 3.4	300	60	5
		Placebo	19	48.2 ± 14.2	29.3 ± 3.7	5.8 ± 3.1			
Sadeghian et al. (40)	Iran	Magnesium oxide	40	41.2 ± 8.8	31.2 ± 5.5	13.2 ± 8.6	250	84	5
		Placebo	40	42.8 ± 8.4	30.9 ± 4.4	12.8 ± 7.5			

^a^Data was presented as mean ± SD. BMI, body mass index.

### Effects of magnesium supplementation on glycemic control

**FIGURE 2:**
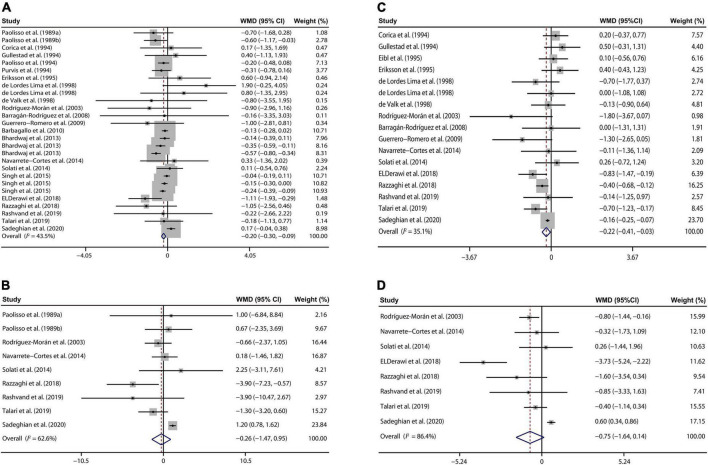
Forest plots for the effects of magnesium supplementation on FPG **(A)**, insulin **(B)**, HbA1c **(C)**, and HOMA-IR **(D)** compared to controls in pooled analysis. For each study, the solid black circles represent the point estimate of the intervention effect. The horizontal line joins the lower and upper limits of the 95% CI of this effect. The open diamonds represent the overall WMD determined with a random-effect model. FPG, fasting plasma glucose; HbA1c, glycated hemoglobin; HOMA-IR, homeostasis model assessment-insulin resistance; WMD, weighted mean difference.

The effect of magnesium supplementation on FPG was reported in 27 observations from 22 studies (7, 20–40). The following analysis revealed that magnesium administration decreased the FPG concentration, with a WMD value of –0.20 mM (95% CI: –0.30, –0.09; *I*^2^ = 43.5%, Figure 2A).

Nine studies (20, 21, 28, 33, 34, 37–40) involving 495 T2D patients presented that oral magnesium had no significant influence on plasma insulin concentration relative to the control group (WMD: –0.26 mU/L; 95% CI: –1.47, 0.95; *I*^2^ = 62.6%, Figure 2B).

Our meta-analysis of 17 interventions (7, 22, 23, 26–30, 33, 34, 36–41) found a significant reduction in HbA1c of T2D populations received magnesium addition relative to the control treatment (WMD: –0.22%; 95% CI: –0.41, –0.03; *I*^2^ = 35.1%, Figure 2C).

We also explored whether magnesium administration regulates insulin sensitivity through analyzing the HOMA-IR data from the 8 studies (28, 33, 34, 36–40). However, no significant difference in HOMA-IR was found between the intervention and control group (WMD: –0.75; 95% CI: –1.64, 0.14; *I*^2^ = 86.4%, Figure 2D).

### Effects of magnesium supplementation on lipid metabolism

**FIGURE 3:**
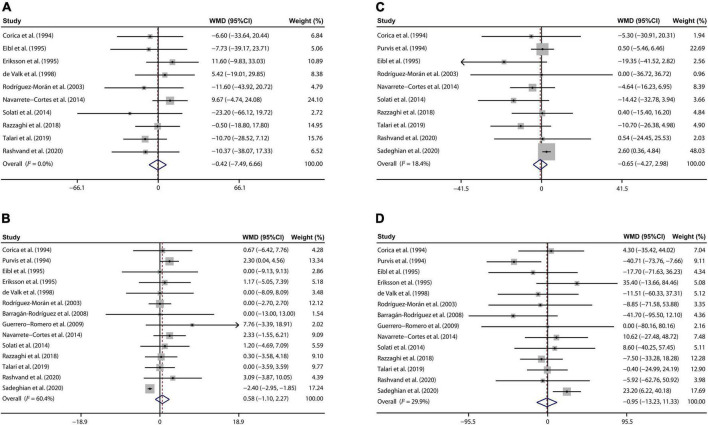
Forest plots for the effects of magnesium supplementation on TC **(A)**, HDL-C **(B)**, LDL-C **(C)**, and TG **(D)** compared to controls in pooled analysis. For each study, the solid black circles represent the point estimate of the intervention effect. The horizontal line joins the lower and upper limits of the 95% CI of this effect. The open diamonds represent the overall WMD determined with a random-effect model. HDL-C, high-density lipoprotein cholesterol; LDL-C, low-density lipoprotein cholesterol; TC, total cholesterol; TG, total triglyceride; WMD, weighted mean difference.

Ten eligible publications (22, 26–28, 33, 34, 37, 39, 41, 42) suggested that there was no remarkable decrease in TC of T2D cases between the magnesium and control groups (WMD: –0.42 mg/dL; 95% CI: –7.49, 6.66; *I*^2^ = 0%, Figure 3A).

Our meta-analysis of 14 RCTs (22, 25–30, 33, 34, 37, 39–42) demonstrated that magnesium treatment had no significant effects on serum HDL-C concentrations than those with placebo treatment (WMD: 0.58 mg/dL; 95% CI: –1.10, 2.27; *I*^2^ = 60.4%, Figure 3B).

There were 10 studies reporting the influence of magnesium addition on serum LDL-C levels (22, 25, 28, 33, 34, 37, 39–42). Further analysis indicated that no prominent changes were found in LDL-C concentrations of T2D patients after magnesium administration than the control group (WMD: –0.65 mg/dL; 95% CI: –4.27, 2.98; *I*^2^ = 18.4%, Figure 3C).

At last, 14 studies (22, 25–30, 33, 34, 37, 39–42) involving 776 T2D persons demonstrated that circulating TG concentrations were not disturbed by the magnesium intervention (WMD: –0.95 mg/dL; 95% CI: –13.23, 11.33; *I*^2^ = 29.9%, Figure 3D).

### Effects of magnesium supplementation on blood pressure

**FIGURE 4:**
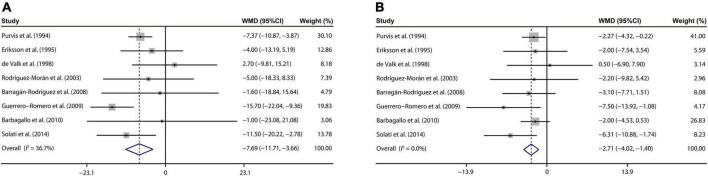
Forest plots for the effects of magnesium supplementation on SBP **(A)** and DBP **(B)** compared to controls in pooled analysis. For each study, the solid black circles represent the point estimate of the intervention effect. The horizontal line joins the lower and upper limits of the 95% CI of this effect. The open diamonds represent the overall WMD determined with a random-effect model. DBP, diastolic blood pressure; SBP, systolic blood pressure; WMD, weighted mean difference.

Our analysis of 8 eligible publications (25–31, 34) demonstrated that magnesium treatment contributed to reducing the SBP, with a WMD value of –7.69 mmHg (95% CI: –11.71, –3.66; *I*^2^ = 36.7%, Figure 4A). On the other hand, a meta-analysis of 8 studies (25–31, 34) indicated that DBP of T2D patient was prominently decreased by the magnesium intervention (WMD: –2.71 mmHg; 95% CI: –4.02, –1.40; *I*^2^ = 0%, Figure 4B).

### Subgroup analyses of magnesium supplementation effects on glycemic control

Subgroup analyses about the use of magnesium for 4 glycemic indicators presented that magnesium treatment in patients with hypomagnesemia (plasma magnesemia ≤0.74 mM) (*P* = 0.020) or for a duration of ≥ 90 d (*P* = 0.013) exhibited a stronger effect on reducing FPG of T2D cases than respective other subgroups (Table 2).

**TABLE 2:** Subgroup analyses for the effects of magnesium supplementation on glycemic, lipid and blood pressure parameters in type 2 diabetes mellitus patients^a^.

Parameter	Subgroup	*n*	WMD (95% CI)	*I*^2^ (%)	*P*-value^b^	*P*-value^c^
FPG	Age, years					
	≤60	11	–0.12 (–0.47, 0.23)	40.9	0.487	0.056
	>60	9	–0.18 (–0.31, –0.05)	0.0	0.006	
	Baseline BMI, kg/m^2^					
	≤30	14	–0.20 (–0.49, 0.09)	10.4	0.174	0.122
	>30	4	–0.15 (–0.59, 0.28)	64.9	0.487	
	Region					
	America	7	–0.17 (–0.65, 0.31)	4.7	0.480	0.984
	Asia	12	–0.20 (–0.35, –0.06)	67.9	0.007	
	Europe	8	–0.17 (–0.31, –0.04)	0.0	0.010	
	Baseline magnesium level, mM					
	≤0.74	14	–0.23 (–0.35, –0.10)	54.3	<0.001	0.020
	>0.74	10	0.00 (–0.16, 0.16)	1.4	0.983	
	Baseline HbA1c					
	≤8	6	0.16 (–0.04, 0.36)	0.0	0.121	0.063
	>8	10	–0.26 (–0.85, 0.33)	33.6	0.385	
	Duration of diabetes, years					
	≤10	11	–0.22 (–0.54, 0.11)	28.2	0.194	0.071
	>10	6	0.11 (–0.09, 0.31)	0.0	0.276	
	Magnesium formulation					
	Inorganic	17	–0.18 (–0.31, –0.05)	54.6	0.008	0.715
	Organic	9	–0.25 (–0.45, –0.06)	17.7	0.011	
	Dosage of intervation, mg/d					
	<300	6	–0.47 (–1.01, 0.06)	71.5	0.079	0.202
	300–399	14	–0.20 (–0.29, –0.11)	33.0	<0.001	
	≥400	7	0.16 (–0.61, 0.94)	0.0	0.683	
	Duration of intervation, d					
	<30	5	–0.15 (–0.31, 0.00)	26.5	0.050	0.013
	30–89	11	–0.12 (–0.28, 0.04)	42.5	0.129	
	≥90	11	–0.34 (–0.58, –0.11)	31.4	0.004	
Insulin	Age, years					
	≤60	6	0.03 (–1.19, 1.25)	62.5	0.961	0.053
	>60	3	–1.15 (–4.66, 2.35)	53.5	0.520	
	Baseline BMI, kg/m^2^					
	≤30	5	–1.33 (–2.76, 0.10)	22.5	0.068	< 0.001
	>30	3	1.13 (0.73, 1.53)	0.0	<0.001	
	Region					
	America	2	–0.22 (–1.41, 0.96)	0.0	0.712	0.159
	Asia	5	–0.81 (–3.13, 1.51)	76.7	0.673	
	Europe	2	0.71 (–2.11, 3.53)	0.0	0.621	
	Baseline magnesium level, mM					
	≤0.74	1	–0.66 (–2.37, 1.05)	–	0.450	0.072
	>0.74	7	–0.37 (–1.93, 1.19)	66.9	0.639	
	Baseline HbA1c					
	≤8	4	0.03 (–1.48, 1.54)	68.8	0.966	0.107
	>8	3	–1.17 (–3.93, 1.60)	55.3	0.408	
	Duration of diabetes, years					
	≤10	5	–4.56 (–7.74, –1.38)	82.9	0.005	< 0.001
	>10	2	–1.02 (–9.85, 7.81)	81.0	0.821	
	Magnesium formulation					
	Inorganic	6	–0.69 (–2.47, 1.08)	75.8	0.443	0.426
	Organic	3	0.31 (–1.11, 1.74)	0.0	0.664	
	Dosage of intervation, mg/d					
	<300	5	–0.43 (–2.41, 1.55)	73.2	0.671	0.079
	300–399	2	0.36 (–1.21, 1.93)	0.0	0.655	
	≥400	2	–0.87 (–2.52, 0.79)	0.0	0.306	
	Duration of intervation, d					
	<30	2	0.71 (–2.11, 3.53)	0.0	0.621	0.220
	30–89	3	–1.66 (–5.90, 2.58)	82.0	0.443	
	≥90	4	–0.43 (–1.42, 0.56)	0.0	0.393	
HbA1c	Age, years					
	≤60	10	–0.42 (–0.73, –0.11)	40.5	0.007	0.933
	>60	6	–0.08 (–0.37, 0.21)	26.2	0.568	
	Baseline BMI, kg/m^2^					
	≤30	14	–0.24 (–0.50, 0.03)	43.5	0.083	0.211
	>30	2	–0.16 (–0.25, –0.07)	0.0	<0.001	
	Region					
	America	6	–0.51 (–1.03, 0.01)	2.1	0.057	0.027
	Asia	6	–0.35 (–0.60, –0.10)	53.7	0.006	
	Europe	5	0.20 (–0.12, 0.51)	0.0	0.226	
	Baseline magnesium level, mM					
	≤0.74	8	–0.37 (–0.83, 0.10)	43.3	0.122	0.403
	>0.74	8	–0.22 (–0.38, –0.06)	21.0	0.009	
	Baseline HbA1c					
	≤8	7	–0.11 (–0.35, 0.13)	30.7	0.358	0.060
	>8	10	–0.35 (–0.66, –0.04)	27.7	0.026	
	Duration of diabetes, years					
	≤10	10	–0.22 (–0.59, 0.16)	47.7	0.26	0.450
	>10	6	–0.14 (–0.32, 0.03)	7.30	0.101	
	Magnesium formulation					
	Inorganic	10	–0.33 (–0.55, –0.11)	33.6	0.003	0.372
	Organic	6	–0.06 (–0.45, 0.34)	40.4	0.782	
	Dosage of intervation, mg/d					
	<300	4	–0.41 (–0.70, –0.13)	69.8	0.005	0.129
	300–399	5	0.17 (–0.19, 0.53)	0.00	0.349	
	≥400	8	–0.20 (–0.63, 0.22)	23.6	0.350	
	Duration of intervation, d					
	30–89	7	–0.18 (–0.26, –0.09)	0.0	<0.001	0.474
	≥90	10	–0.25 (–0.64, 0.14)	51.9	0.214	
HOMA-IR	Age, years					
	≤60	7	–0.66 (–1.59, 0.28)	87.6	0.168	0.070
	>60	1	–1.60 (–3.54, 0.34)	–	0.106	
	Baseline BMI, kg/m^2^					
	≤30	6	–1.13 (–2.09, –0.18)	71.7	0.020	< 0.001
	>30	2	0.41 (–0.31, 1.14)	36.3	0.264	
	Region					
	America	2	–0.72 (–1.30, –0.14)	0.0	0.015	0.001
	Asia	6	–0.85 (–2.06, 0.36)	87.6	0.169	
	Baseline magnesium level, mM					
	≤ 0.74	2	–2.18 (–5.05, 0.69)	91.9	0.136	0.101
	>0.74	6	–0.13 (–0.83, 0.58)	60.9	0.724	
	Baseline HbA1c					
	≤8	4	0.01 (–0.74, 0.77)	64.8	0.978	< 0.001
	>8	4	–1.45 (–2.99, 0.09)	80.3	0.065	
	Duration of diabetes, years					
	≤10	5	–1.07 (–2.14, 0.00)	76.6	0.050	< 0.001
	>10	1	0.60 (0.34, 0.86)	–	<0.001	
	Magnesium formulation					
	Inorganic	6	–0.31 (–1.10, 0.48)	79.5	0.444	0.101
	Organic	2	–2.02 (–5.36, 1.33)	90.4	0.237	
	Dosage of intervation, mg/d					
	<300	4	–1.12 (–2.67, 0.43)	92.4	0.158	0.003
	300–399	2	–0.08 (–1.17, 1.01)	0.00	0.881	
	≥400	2	–0.80 (–1.42, –0.19)	0.00	0.011	
	Duration of intervation, d					
	30–89	3	–0.34 (–1.91, 1.22)	67.1	0.666	< 0.001
	≥90	5	–0.95 (–1.96, 0.05)	77.2	0.062	
TC	Age, years					
	≤60	5	–3.81 (–15.38, 7.75)	20.9	0.518	0.581
	>60	5	1.73 (–8.68, 12.15)	0.0	0.744	
	Baseline BMI, kg/m^2^					
	≤30	9	–3.62 (–11.74, 4.50)	0.0	0.382	0.115
	>30	1	9.67 (–4.74, 24.08)	–	0.188	
	Region					
	America	2	4.16 (–14.11, 22.42)	28.0	0.656	0.244
	Asia	4	–7.68 (–18.87, 3.51)	0.0	0.179	
	Europe	4	2.81 (–9.86, 15.47)	0.0	0.664	
	Baseline magnesium level, mM					
	≤0.74	3	1.53 (–14.01, 17.06)	0.0	0.847	0.783
	>0.74	7	–0.93 (–8.87, 7.02)	0.0	0.819	
	Baseline HbA1c					
	≤8	4	–1.08 (–12.2, 10.03)	16.0	0.849	0.844
	>8	5	1.10 (—9.86, 12.05)	0.0	0.845	
	Duration of diabetes, years					
	≤10	6	–5.85 (–16.31, 4.61)	0.0	0.273	0.583
	>10	2	0.02 (–18.11, 18.15)	0.0	0.999	
	Magnesium formulation					
	Inorganic	5	–8.10 (–18.67, 2.48)	0.0	0.133	0.103
	Organic	4	4.37 (–6.24, 14.99)	0.0	0.419	
	Dosage of intervation, mg/d					
	<300	2	–5.73 (–18.5, 7.03)	0.0	0.379	0.617
	300–399	5	2.11 (–8.04, 12.26)	0.0	0.683	
	≥400	3	1.53 (–14.01, 17.06)	0.0	0.847	
	Duration of intervation, d					
	30–89	3	–4.25 (–17.54, 9.05)	0.0	0.531	0.505
	≥90	7	0.95 (–7.63, 9.53)	3.4	0.828	
TG	Age, years					
	≤60	8	0.76 (–16.71, 18.23)	43.3	0.932	0.259
	>60	6	–5.24 (–21.76, 11.29)	0.0	0.535	
	Baseline BMI, kg/m^2^					
	≤30	10	–0.97 (–13.76, 11.83)	0.0	0.882	0.259
	>30	3	–0.99 (–40.68, 38.71)	82.4	0.961	
	Region					
	America	5	–19.00 (–42.89, 4.90)	19.6	0.119	0.052
	Asia	5	7.70 (–6.42, 21.82)	21.2	0.285	
	Europe	4	3.60 (–19.89, 27.08)	0.0	0.764	
	Baseline magnesium level, mM					
	≤0.74	5	–5.62 (–33.47, 22.23)	13.7	0.692	0.337
	>0.74	8	8.30 (–2.18, 18.78)	0.0	0.121	
	Baseline HbA1c					
	≤8	5	12.34 (0.21, 24.48)	0.0	0.046	0.121
	>8	7	–4.12 (–20.98, 12.75)	0.0	0.633	
	Duration of diabetes, years					
	≤10	6	2.20 (–14.96, 19.36)	0.0	0.801	0.353
	>10	5	2.79 (–20.74, 26.32)	39.5	0.816	
	Magnesium formulation					
	Inorganic	9	–5.11 (–22.22, 12.01)	49.2	0.559	0.857
	Organic	4	–0.42 (–22.31, 21.48)	0.0	0.970	
	Dosage of intervation, mg/d					
	<300	3	7.07 (–12.76, 26.89)	58.1	0.485	0.163
	300–399	6	–8.52 (–26.67, 9.64)	9.20	0.358	
	≥400	5	–5.62 (–33.47, 22.23)	13.7	0.692	
	Duration of intervation, d					
	30-89	6	–8.03 (–31.96, 15.91)	67.8	0.511	0.988
	≥90	8	2.71 (–12.45, 17.87)	0.0	0.726	
HDL-C	Age, years					
	≤60	8	0.78 (–1.45, 3.01)	76.6	0.495	0.091
	>60	6	0.44 (–2.19, 3.06)	0.0	0.744	
	Baseline BMI, kg/m^2^					
	≤30	10	0.54 (–1.01, 2.10)	0.0	0.494	0.102
	>30	3	0.51 (–3.32, 4.33)	90.3	0.795	
	Region					
	America	5	1.61 (0.06, 3.17)	0.0	0.042	< 0.001
	Asia	5	–0.76 (–2.75, 1.24)	43.1	0.458	
	Europe	4	0.60 (–3.10, 4.30)	0.0	0.752	
	Baseline magnesium level, mM					
	≤ 0.74	5	0.49 (–1.81, 2.79)	0.0	0.676	0.103
	>0.74	8	–0.09 (–1.99, 1.81)	46.6	0.929	
	Baseline HbA1c					
	≤8	5	–0.54 (–2.85, 1.78)	50.1	0.649	0.106
	>8	7	0.51 (–1.35, 2.37)	0.0	0.591	
	Duration of diabetes, years					
	≤10	6	0.43 (–1.39, 2.24)	0.0	0.647	0.004
	>10	5	–1.81 (–3.59, –0.03)	7.70	0.047	
	Magnesium formulation					
	Inorganic	9	0.43 (–1.62, 2.47)	70.0	0.682	0.128
	Organic	4	1.48 (–1.49, 4.45)	0.0	0.329	
	Dosage of intervation, mg/d					
	<300	3	–1.48 (–3.34, 0.38)	41.6	0.119	< 0.001
	300–399	6	2.07 (0.37, 3.77)	0.0	0.017	
	≥400	5	0.49 (–1.81, 2.79)	0.0	0.676	
	Duration of intervation, d					
	30–89	6	0.32 (–2.49, 3.12)	74.9	0.825	0.101
	≥90	8	0.76 (–0.88, 2.40)	0.0	0.365	
LDL-C	Age, years					
	≤60	7	0.22 (–3.30, 3.74)	17.9	0.902	0.193
	>60	3	–6.10 (–17.69, 5.50)	1.3	0.303	
	Baseline BMI, kg/m^2^					
	≤30	7	–7.75 (–15.43, –0.07)	0.0	0.048	0.015
	>30	3	2.12 (0.06, 4.18)	0.0	0.044	
	Region					
	America	3	–0.56 (–5.81, 4.69)	0.0	0.833	0.146
	Asia	5	–1.58 (–8.35, 5.18)	32.4	0.646	
	Europe	2	–13.33 (–30.09, 3.43)	0.0	0.119	
	Baseline magnesium level, mM					
	≤0.74	2	–14.18 (–33.16, 4.80)	0.0	0.143	0.102
	>0.74	7	–1.06 (–5.87, 3.76)	19.5	0.667	
	Baseline HbA1c					
	≤8	5	–3.95 (–11.8, 3.89)	49.4	0.323	0.225
	>8	3	–5.34 (—16.72, 6.05)	0.0	0.358	
	Duration of diabetes, years					
	≤10	5	–10.94 (–20.30, –1.57)	0.0	0.022	0.006
	>10	2	2.54 (0.31, 4.77)	0.0	0.025	
	Magnesium formulation					
	Inorganic	7	1.63 (–0.74, 4.00)	2.60	0.177	0.061
	Organic	3	–7.45 (–16.99, 2.08)	0.0	0.126	
	Dosage of intervation, mg/d					
	<300	3	0.53 (–5.76, 6.81)	27.7	0.870	0.093
	300–399	5	–1.69 (–6.59, 3.21)	0.0	0.499	
	≥400	2	–14.18 (–33.16, 4.80)	0.0	0.143	
	Duration of intervation, d					
	30-89	5	2.25 (0.19, 4.31)	0.0	0.033	0.004
	≥90	5	–9.29 (–16.90, –1.67)	0.0	0.017	
SBP	Age, years					
	≤60	4	–10.25 (–15.02, –5.47)	47.6	<0.001	0.032
	>60	4	–1.59 (—8.09, 4.92)	0.0	0.632	
	Baseline BMI, kg/m^2^					
	≤30	5	–7.91 (–14.43, –1.40)	57.1	0.017	0.392
	>30	1	–7.37 (–10.87, –3.87)	–	<0.001	
	Region					
	America	4	–9.22 (–14.94, –3.49)	51.4	0.002	0.125
	Asia	1	–11.5 (–20.22, –2.78)	–	0.010	
	Europe	3	–1.59 (–8.61, 5.44)	0.0	0.658	
	Baseline magnesium level, mM					
	≤0.74	5	–7.81 (–14.67, –0.95)	41.5	0.026	0.480
	>0.74	2	–5.14 (–18.98, 8.70)	70.0	0.467	
	Duration of diabetes, years					
	≤10	3	–7.41 (–13.12, –1.69)	0.0	0.011	0.377
	>10	3	–6.06 (–19.33, 7.22)	74.6	0.371	
	Magnesium formulation					
	Inorganic	5	–9.71 (–14.13, –5.28)	38.3	<0.001	0.046
	Organic	2	1.80 (–9.09, 12.69)	0.0	0.746	
	Dosage of intervation, mg/d					
	300–399	4	–6.86 (–11.26, –2.46)	17.8	0.002	0.269
	≥400	4	–8.24 (–15.8, –0.67)	51.4	0.033	
	Duration of intervation, d					
	30–89	3	–7.00 (–10.39, –3.61)	0.0	<0.001	0.314
	≥90	5	–7.91 (–14.43, –1.40)	57.1	0.017	
DBP	Age, years					
	≤60	4	–3.94 (–6.60, –1.28)	31.2	0.004	0.366
	>60	4	–2.02 (–4.01, –0.04)	0.0	0.046	
	Baseline BMI, kg/m^2^					
	≤30	5	–4.08 (–6.85, –1.31)	5.90	0.004	0.280
	>30	1	–2.27 (–4.32, –0.22)	–	0.030	
	Region					
	America	4	–2.77 (–4.52, –1.03)	0.0	0.002	0.214
	Asia	1	–6.31 (–10.88, –1.74)	–	0.007	
	Europe	3	–1.78 (–3.98, 0.42)	0.0	0.113	
	Baseline magnesium level, mM					
	≤0.74	5	–2.68 (–4.58, –0.78)	0	0.006	0.428
	>0.74	2	–3.55 (–10.10, 3.00)	57.6	0.288	
	Duration of diabetes, years					
	≤10	3	–4.15 (–7.35, –0.95)	0.0	0.011	0.803
	>10	3	–3.55 (–7.50, 0.40)	24.4	0.078	
	Magnesium formulation					
	Inorganic	5	–3.40 (–5.24, –1.57)	8.40	<0.001	0.314
	Organic	2	–1.74 (–4.13, 0.66)	0	0.155	
	Dosage of intervation, mg/d					
	300–399	4	–2.54 (–4.23, –0.86)	14.3	0.003	0.516
	≥400	4	–3.56 (–6.43, –0.69)	0.0	0.015	
	Duration of intervation, d					
	30–89	3	–2.26 (–3.77, –0.76)	0.0	0.003	0.234
	≥90	5	–4.08 (–6.85, –1.31)	5.9	0.004	

^a^DBP, diastolic blood pressure; FPG, fasting plasma glucose; HbA1c, glycated hemoglobin; HDL-C, high-density lipoprotein cholesterol; HOMA-IR, homeostasis model assessment of insulin resistance; TC, total cholesterol; TG, total triglycerides; LDL-C, low-density lipoprotein cholesterol; SBP, systolic blood pressure; WMD, weighted mean difference.

^b^*P* values for heterogeneity within each subgroup.

^c^*P* values for subgroup differences between groups.

As shown in Table 2, the influences of magnesium addition on insulin concentration were stronger among >30 than those BMI ≤ 30 kg/m^2^ (*P* < 0.001). In addition, T2D person whose duration of diabetes ≤10 years had a greater decline in circulating insulin concentration after magnesium supplementation than that had a longer course of disease (*P* < 0.001).

Compared with the American and European populations, our analysis revealed that magnesium administration exerted a significant effect on HbA1c in Asian T2D persons (*P* = 0.027, Table 2). However, the other factors were not significant determinants of between-study heterogeneity for HbA1c change during the magnesium treatment (*P* > 0.05).

Subgroup analyses revealed that magnesium addition at ≥ 400 mg/d dosage (*P* = 0.003) or for ≥90 d duration (*P* < 0.001) had a greater effect on HOMA-IR in T2D persons those are from America (*P* = 0.001), or with BMI ≤ 30 kg/m^2^ (*P* < 0.001), or with better baseline glycemic control (HbA1c > 8, *P* < 0.001) or with diabetes ≤ 10 years (*P* < 0.001) compared with the respective subgroups (Table 2).

### Subgroup analyses of magnesium supplementation effects on lipid metabolism

Subgroup analyses based on the human health status and operational details of intervention revealed no significant differences in the influences of magnesium intervention on circulating TC and TG concentrations in T2D cases (*P* > 0.05, Table 2).

As shown in Table 2, our analysis revealed that magnesium application at a dosage of 300–399 mg/d (*P* < 0.001) exerted a more positive effect on increasing the serum HDL-C concentrations in T2D patients those were from America (*P* < 0.001), or with diabetes ≤10 years (*P* = 0.004). Subjects with BMI ≤ 30 kg/m^2^ (*P* = 0.015) or diagnosed as diabetes less than 10 years (*P* = 0.006) had lower plasma LDL-C concentrations after magnesium treatment. Furthermore, duration of administration is also a potential source of heterogeneity for the influences of magnesium on LDL-C variety (*P* = 0.004).

### Subgroup analyses of magnesium supplementation effects on blood pressure

The effect of magnesium addition on SBP was greater for subject’s age ≤60 than those age > 60 years (*P* = 0.032). Besides, the effect of magnesium intervention on SBP was stronger for inorganic supplements than for organic supplements (*P* = 0.046). However, factors about the human baseline metabolic status and mode of intervention had no effects on the influence of magnesium on DBP (*P* > 0.05).

### Dose-effect analyses

**FIGURE 5:**
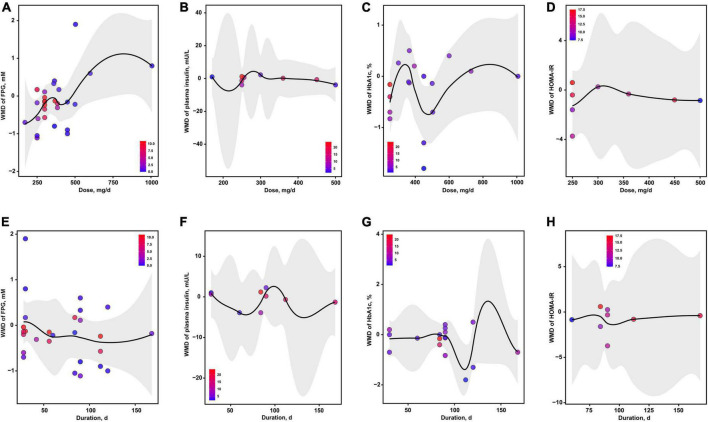
The effects of dosage or duration of magnesium intervention on glycemic control for FPG **(A,E)**, insulin **(B,F)**, HbA1c **(C,G)**, and HOMA-IR **(D,H)**, respectively. FPG, fasting plasma glucose; HbA1c, glycated hemoglobin; HOMA-IR, homeostasis model assessment-insulin resistance.

The dose-effect analyses showed that the optimal dosages of magnesium addition for FPG, insulin, HbA1c and HOMA-IR were 171, 218, 476 and 250 mg/d, respectively (Figures 5A–D). The optimal durations of magnesium administration for FPG, insulin, HbA1c and HOMA-IR were 124, 132, 111 and 95 d, respectively (Figures 5E–H).

**FIGURE 6:**
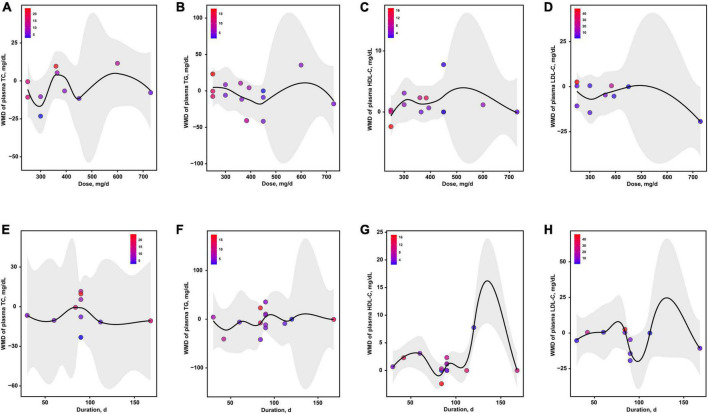
The effects of dosage or duration of magnesium intervention on lipid metabolism for TC **(A,E)**, TG **(B,F)**, HDL-C **(C,G)**, and LDL-C **(D,H)**, respectively. HDL-C, high-density lipoprotein cholesterol; LDL-C, low-density lipoprotein cholesterol; TC, total cholesterol; TG, total triglyceride.

The dose-effects of magnesium intervention on 4 serum lipids indicators were shown in Figure 6. The optimal dosages of magnesium supplementation that may mediate TC, TG, HDL-C and LDL-C were 300, 438, 250 and 729 mg/d, respectively (Figures 6A–D). In addition, the optimal durations of magnesium supplementation that may mediate TC, TG, HDL-C and LDL-C were 128, 46, 81 and 98 d, respectively (Figures 6E–H).

**FIGURE 7:**
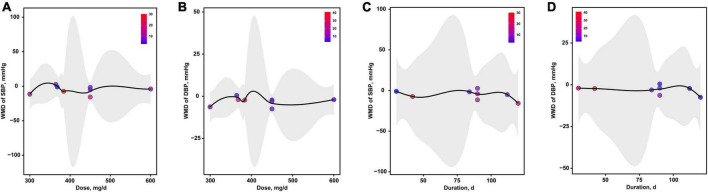
The effects of dosage or duration of magnesium intervention on blood pressure for SBP **(A,C)** and DBP **(B,D)**, respectively. DBP, diastolic blood pressure; SBP, systolic blood pressure.

As presented in Figure 7, the optimal dosages of magnesium addition for SBP and DBP were both 300 mg/d, respectively (Figures 7A,B). The optimal durations of magnesium supplementation for SBP and DBP were both 120 d, respectively (Figures 7C,D).

### Publication bias and sensitivity analysis

As shown in Table 3 and Supplementary Figures 1–3, no significant publication bias were found for the effects of intervention on FPG, insulin, HbA1c, HOMA-IR, SBP and DBP (all *P* > 0.05). For the effects of magnesium supplementation on improving the levels of serum lipids, significant publication bias were found for HDL-C and LDL-C (*P* = 0.001 and *P* = 0.008, respectively), but not for TC and TG (*P* = 0.109 and *P* = 0.142, respectively). Furthermore, sensitivity analyses showed that no single study had an effect on the pooled effect size (Supplementary Figures 4–6).

**TABLE 3:** Publication bias examined by Egger’s linear regression test^a^.

Parameters	*P* for Egger’s test
**Glycemic indicators**	
FPG	0.662
Insulin	0.085
HbA1c	0.619
HOMA-IR	0.082
**Lipid indicators**	
TC	0.109
TG	0.142
HDL-C	0.001
LDL-C	0.008
**Blood pressure indicators**	
SBP	0.403
DBP	0.454

^a^DBP, diastolic blood pressure; FPG, fasting plasma glucose; HbA1c, glycated hemoglobin; HDL-C, high-density lipoprotein cholesterol; HOMA-IR, homeostasis model assessment-insulin resistance; LDL-C, low-density lipoprotein cholesterol; SBP, systolic blood pressure; TC, total cholesterol; TG, total triglyceride.

## Discussion

We analyzed the data from 24 RCTs with 1,325 cases across 11 countries, which offered the most up-to-date evidence demonstrating the effects and operational details of oral magnesium on improving hyperglycemia, hypercholesterolemia, and hypertension in T2D patients.

The increased prevalence of hypomagnesaemia identified in diabetic cases informs the design and development of magnesium supplementation to ameliorate the status of T2D patients (43). Similar to previous review that magnesium supplementation for 1 to 4 months reduced FPG (14), and contrary to the meta-analysis done by Chua et al. (44) that routine magnesium intervention had no effects on HbA1c, our updated findings revealed that oral magnesium both significantly decreased the FPG and increased the HbA1c in T2D persons, highlighting the important role of magnesium in improving the short- and long-term glycemic control. Magnesium may improve glucose metabolism via several pathways. One possible explanation for this observation is that Mg^2+^ may adjust for the rate of glucokinase activity and then regulated the glucose utilization (45). In addition, binding of MgATP, an adenine nucleotide, to the sulfonylurea receptor 1 affects the opening of the ATP-sensitive K^+^ channel that controls the membrane depolarization and subsequent exocytosis of insulin-containing granules (45), which further mediated the circulating glucose concentration. At last, it is established that magnesium may play key roles on other parameters closely related to glucose metabolism, including body composition, general health, and sleep quality (46). Further subgroup analysis showed that baseline magnesium concentration is a main factor contributing to the heterogeneity on the effects of magnesium on FPG. Blaine et al. (47) pointed out that higher magnesium supply rapidly elevates its renal output, suggesting that basal magnesium status may be associated with the efficacy of magnesium administration. Previous work indicated that magnesium deficiency decreased insulin sensitivity throughout blocking insulin pathways to trigger the acute phaseresponse (48). Although our overall ungrouped analysis showed no significant effects of magnesium on HOMA-IR, subgroup analyses demonstrated that patient’s health status and mode of intervention are both the remarkable determinants of heterogeneity, suggesting that the clinical application of magnesium addition for increasing insulin sensitivity should be flexible specific to each person’s settings.

Subgroup analysis in current study showed that magnesium administration at 300-399 mg/d dosage led to an increase in plasma HDL-C concentrations in patients with T2D from America, which was supported by prevenient research that the beneficial effect of magnesium on dyslipidemia partly resulted from the generation of HDL by depressing HMG-CoA reductase and stimulation of lecithin cholesterol acyl transferase (49). On the other hand, a recent study showed that magnesium supplementation reduced lipid deposition in hepatocytes by activating autophagy by activating the AMPK-mTOR pathway, indicating a relationship between magnesium and lipid accumulation (50). Furthermore, Sales et al. (51) observed that the increased insulin sensitivity occurred after magnesium addition in our current study may also help to improve the dyslipidemia. The present results showed that magnesium supplementation also lessened the hyperglycemia of T2D patients whose baseline BMI ≤ 30 kg/m^2^ or durations of diabetes ≤10 years through decreasing the plasma LDL-C concentrations. Ample evidence indicated that dietary intake of divalent cations including magnesium promoted fecal excretion of fat (52), implying that the reduced serum LDL-C after magnesium intervention may be in part due to the inhibition of absorption. It is noteworthy that magnesium addition may also increase serum LDL-C concentrations in obese T2D cases with longer duration of diabetes, which implied that the clinical application of magnesium supplementation in relieving hyperglycemia should be performed in early stage of diabetes specific to each patient’s metabolic status.

Contrary to the study done by Song et al. (14) and in line with previous findings reported by Asbaghi et al. (53) and Zhang et al. (54), our pooled results with a greater number of RCTs proved that magnesium supplementation induced a profound decline in blood pressure of T2D patients. Wu et al. (55) further found that every 0.1 mmol/L increment in circulating serum magnesium level was associated with a 4% reduction in hypertension incidence. Accumulating evidence illustrated that magnesium released intracellular sodium and calcium stores through triggering membrane -Na^+^/K^+^ -ATPase and thereby decreases the blood hypertension through reduces the peripheral vascular resistance (56). Secondly, magnesium reduces hypertension may also attribute to its influences on the abundance of osteopontin, matrix Gla protein, and receptor potential melastin 7 (TRPM7), which were collectively observed to depress the vascular calcification (57). Thirdly, magnesium can also decrease vascular tone by releasing the nitric oxide (NO) from the coronary endothelium as well as resisting the influences of vasoconstrictor molecules such as calcium, bradykinin, or serotonin (58). Similar to previous research by Asbaghi et al. (53), our data exhibited that supplementation with inorganic magnesium had more positive effects on reducing blood pressure than the organic formulation. Magnesium is absorbed by both passive diffusion and active transport (59). Given that lower magnesium intakes may elevate the role of active process, it is possible that the amount of magnesium intake may regulate the magnesium absorption from compounds with different properties (60). However, the bioavailability of magnesium from inorganic and organic supplements, including absorption, retention or urinary excretion, needs to be further assessed.

With respect to the contradictory results from former trials, our updated meta-analysis increased the statistical power to systematically examine the effects of oral magnesium on indicators of glycemic control, lipid metabolism and blood pressure that related to complications in T2D individuals (61, 62). On this foundation, we followed subgroup and dose-effect analyses to explore the potential factors influencing the effects of magnesium administration, which provided a key reference for clinical application of this strategy in T2D patients. However, several limitations merit consideration. First, some of the included publications had small groups, such as <30 persons/group). Second, magnesium formulation, dosage and duration varied across different RCTs, which induced differential results and resulted in difficulties in assessing the real effect of magnesium supplementation. Third, some RCTs offered limited consideration to alimentary magnesium intake that may influence the effects of magnesium treatment. Nevertheless, the likelihood of this bias has been evaluated through the subgroup analysis based on the baseline circulating magnesium levels.

## Conclusion

In summary, our findings indicated that magnesium supplementation had profitable effects on serum glucose, lipids, and pressure controls. Subgroup analyses revealed that magnesium administration in patients with hypomagnesemia or for a duration of ≥90 d exhibited a stronger effect on decreasing FPG, while intervention at ≥400 mg/d dosage or for ≥90 d duration had a greater effect on HOMA-IR in T2D persons with BMI ≤ 30 kg/m^2^ or baseline HbA1c > 8% or duration of diabetes ≤10 years. Subjects with BMI ≤ 30 kg/m^2^ or diagnosed as diabetes less than 10 years had lower plasma LDL-C concentrations, and American T2D patients received a dosage of 300–399 mg/d had greater HDL-C concentrations after oral magnesium supplementation. In addition, the inorganic magnesium supplements were more beneficial for lowering the SBP of younger T2D populations. At last, 279 mg/d for 116 d, 429 mg/d for 88 d and 300 mg/120 d are the average optimal dosage and duration for improving glycemic, circulating lipids, and blood pressure controls, respectively. Taken together, our study provide clinically relevant information on the adjuvant therapy of magnesium for T2D. In the future, guidelines for clinical practice of magnesium supplementation including dosage and duration according to each individual’s health status as well as the chronic safety of magnesium addition at high dosages should be further assessed in large multinational prospective RCTs, and the causal effects would be explored (3, 63–67).

## Data availability statement

The original contributions presented in the study are included in the article/Supplementary material, further inquiries can be directed to the corresponding author.

## Author contributions

MX conceived this study and supervised this research. LX and XL conceived the study, searched the literature, and performed data extraction. XL conducted the primary statistical analysis. XW provided statistical expertise. LX wrote the manuscript. LX, MX, and XW contributed by critically revising the manuscript. All authors read and approved the final manuscript.
