# Supplementary material for: Effects of magnesium supplementation on improving hyperglycemia, hypercholesterolemia, and hypertension in type 2 diabetes: A pooled analysis of 24 randomized controlled trials
Source: Front Nutr. 2023 Jan 18;9:1020327. doi: 10.3389/fnut.2022.1020327 (PMC9889557; doi:10.3389/fnut.2022.1020327)
Supplement: Supplementary file 1 [file Data_Sheet_1.docx]

Supplementary Material

1 Supplementary Figures

**
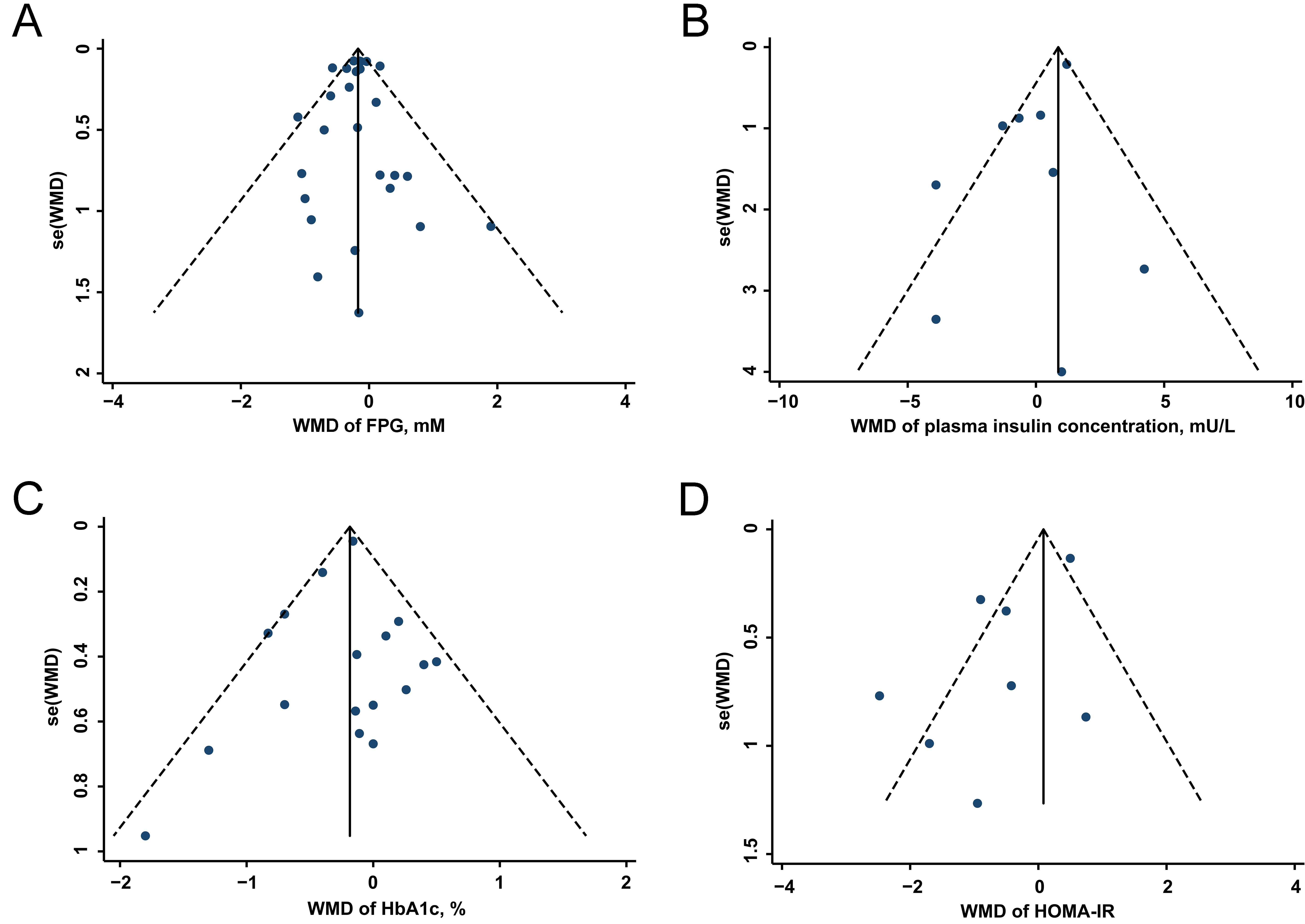
**

**Supplemental Figure 1.** Funnel plots for the included studies about the effects of magnesium supplementation on FPG (A), insulin (B), HbA1c (C) and HOMA-IR (D) in type 2 diabetes mellitus patients. FPG, fasting plasma glucose; HbA1c, glycated hemoglobin; HOMA-IR, homeostasis model assessment of insulin resistance.

**
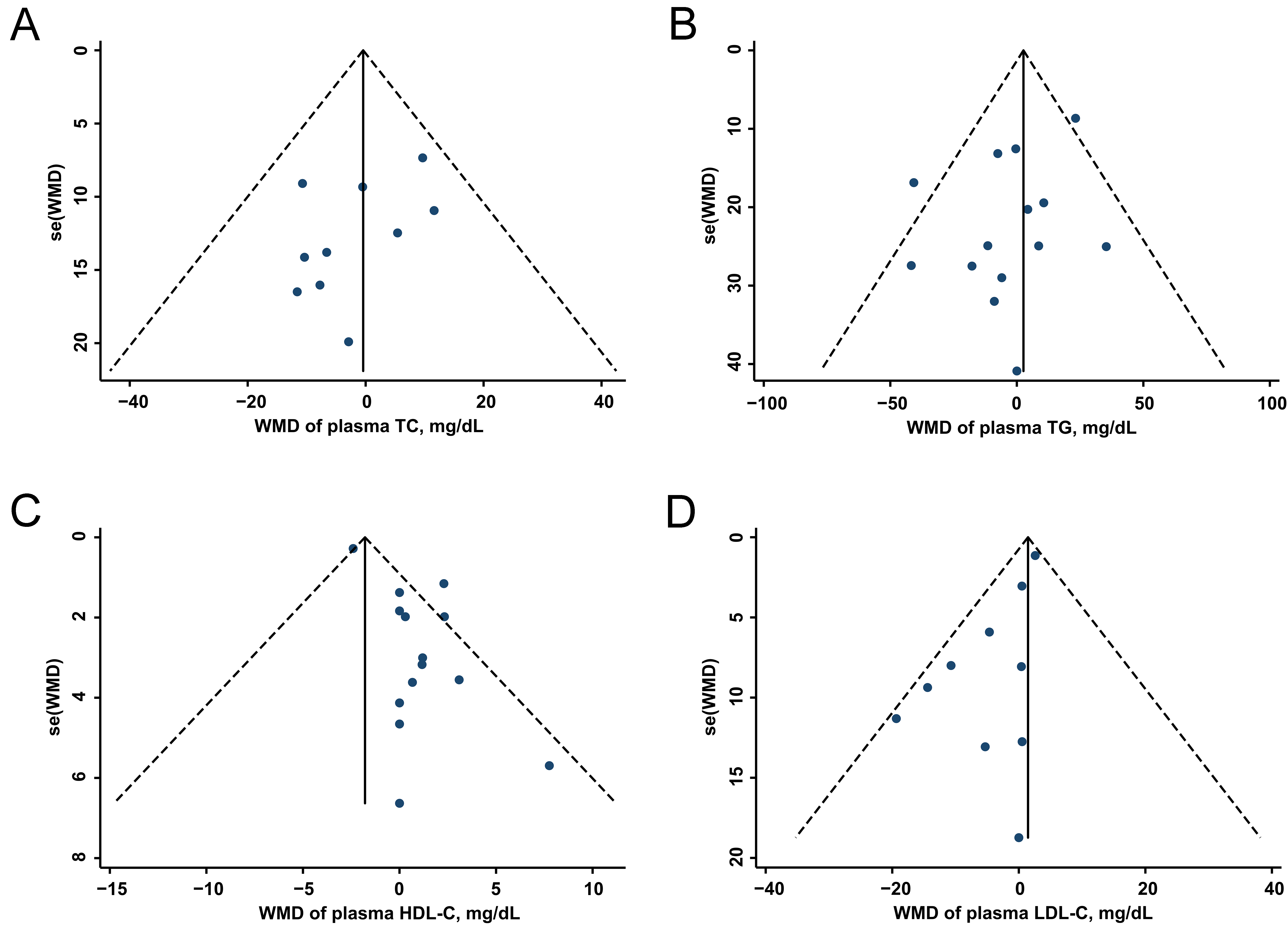
**

**Supplemental Figure 2.** Funnel plots for the included studies about the effects of magnesium supplementation on TC (A), TG (B), HDL-C (C) and LDL-C (D) in type 2 diabetes mellitus patients. HDL-C, high-density lipoprotein cholesterol; TC, total cholesterol; TG, triglycerides; LDL-C, low-density lipoprotein cholesterol.

**
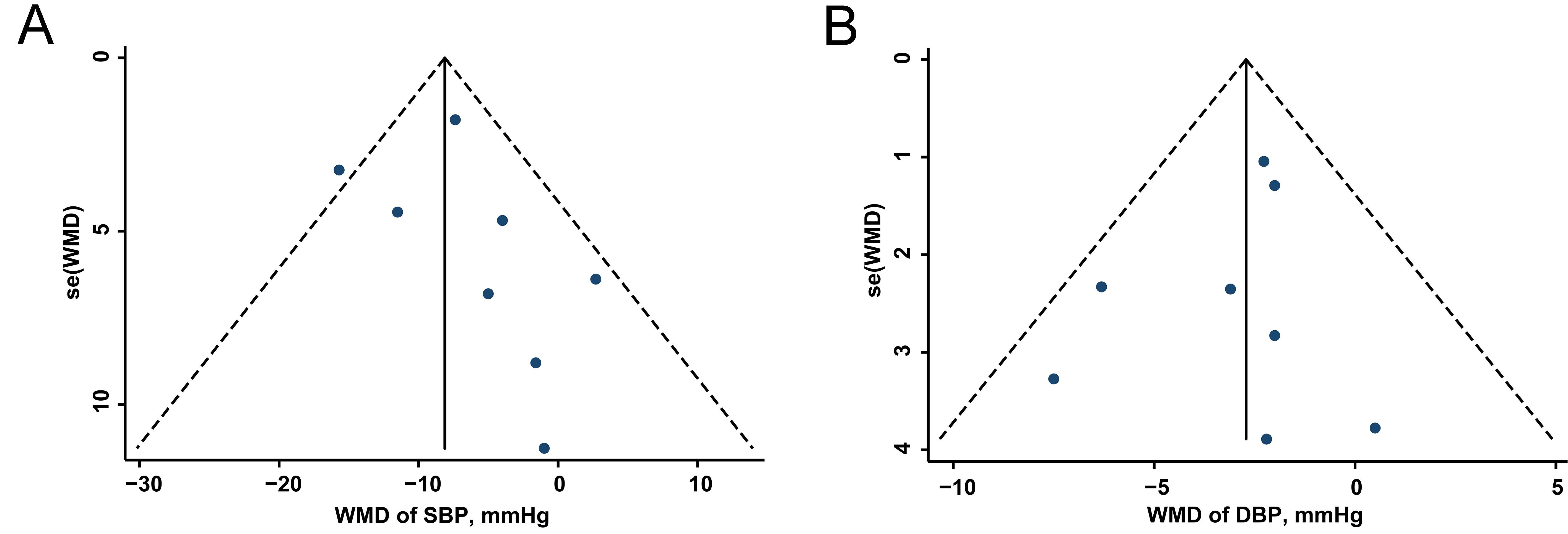
**

**Supplemental Figure 3.** Funnel plots for the included studies about the effects of magnesium supplementation on SBP (A) and DBP (B) in type 2 diabetes mellitus patients. DBP, diastolic blood pressure; SBP, systolic blood pressure.

**
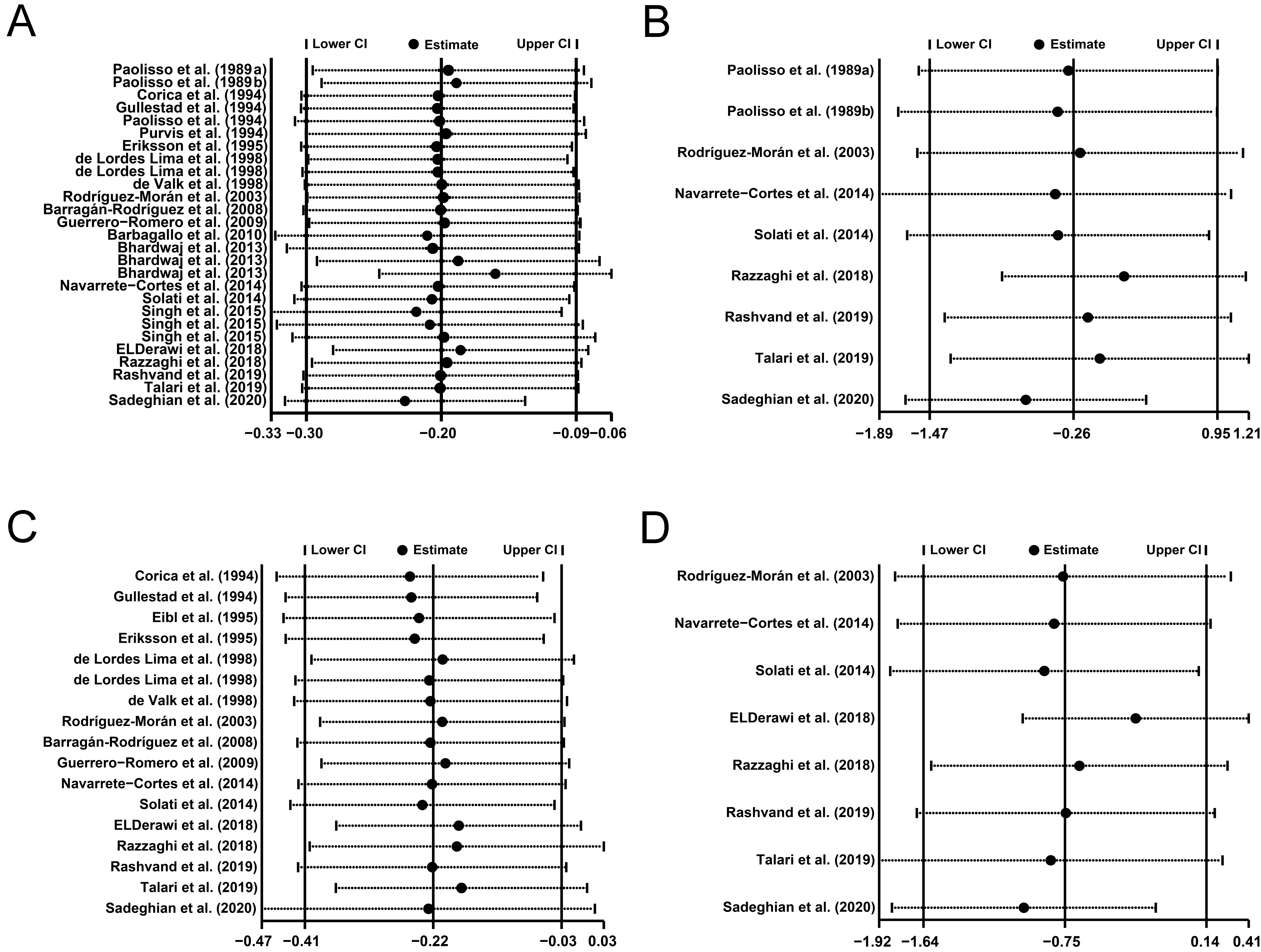
**

**Supplemental Figure 4.** Sensitivity analysis for the included studies about the effects of magnesium supplementation on FPG (A), insulin (B), HbA1c (C) and HOMA-IR (D) in type 2 diabetes mellitus patients. FPG, fasting plasma glucose; HbA1c, glycated hemoglobin; HOMA-IR, homeostasis model assessment of insulin resistance.


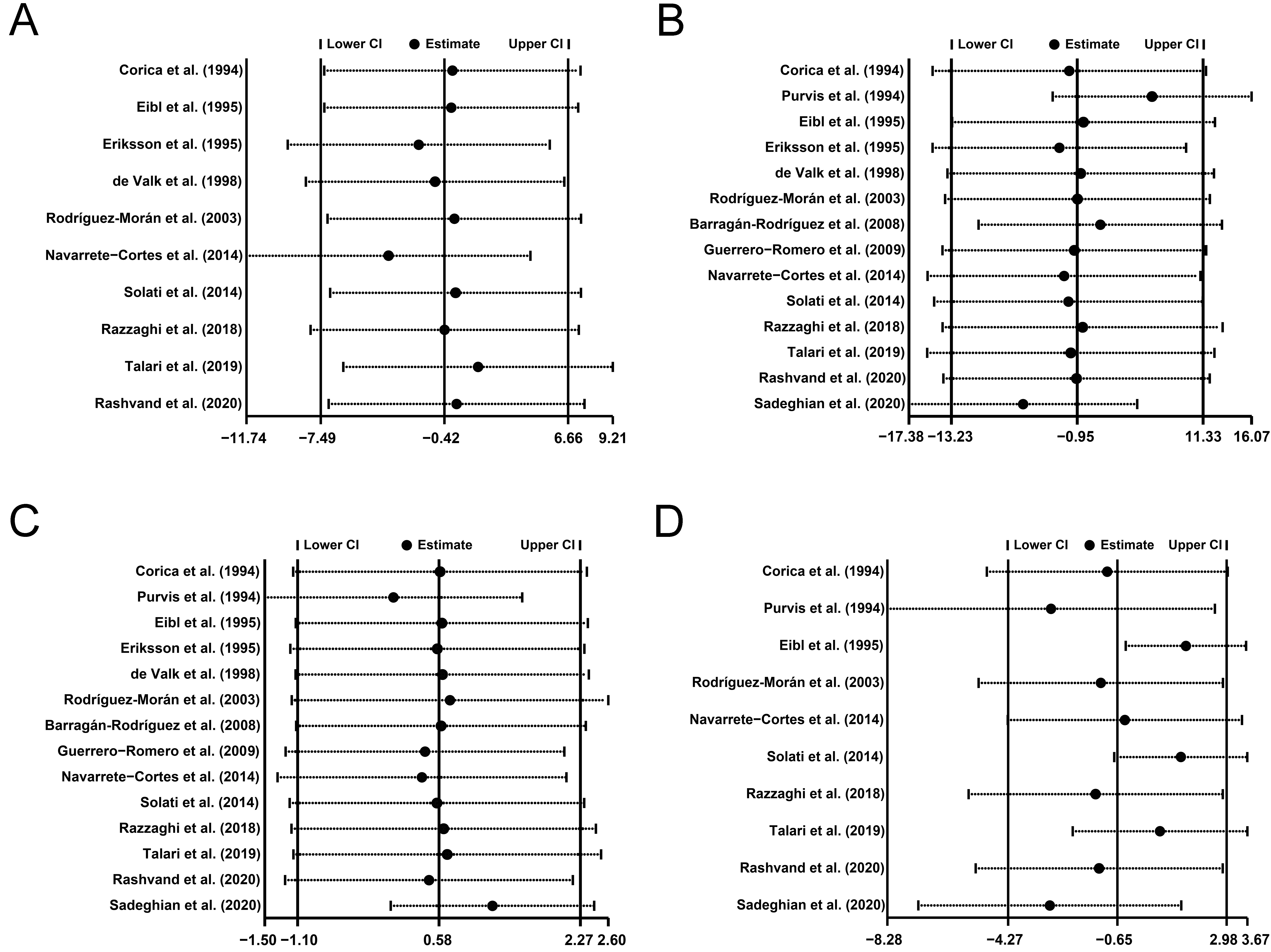


**Supplemental Figure 5.** Sensitivity analysis for the included studies about the effects of magnesium supplementation on TC (A), TG (B), HDL-C (C) and LDL-C (D) in type 2 diabetes mellitus patients. HDL-C, high-density lipoprotein cholesterol; TC, total cholesterol; TG, triglycerides; LDL-C, low-density lipoprotein cholesterol.

**
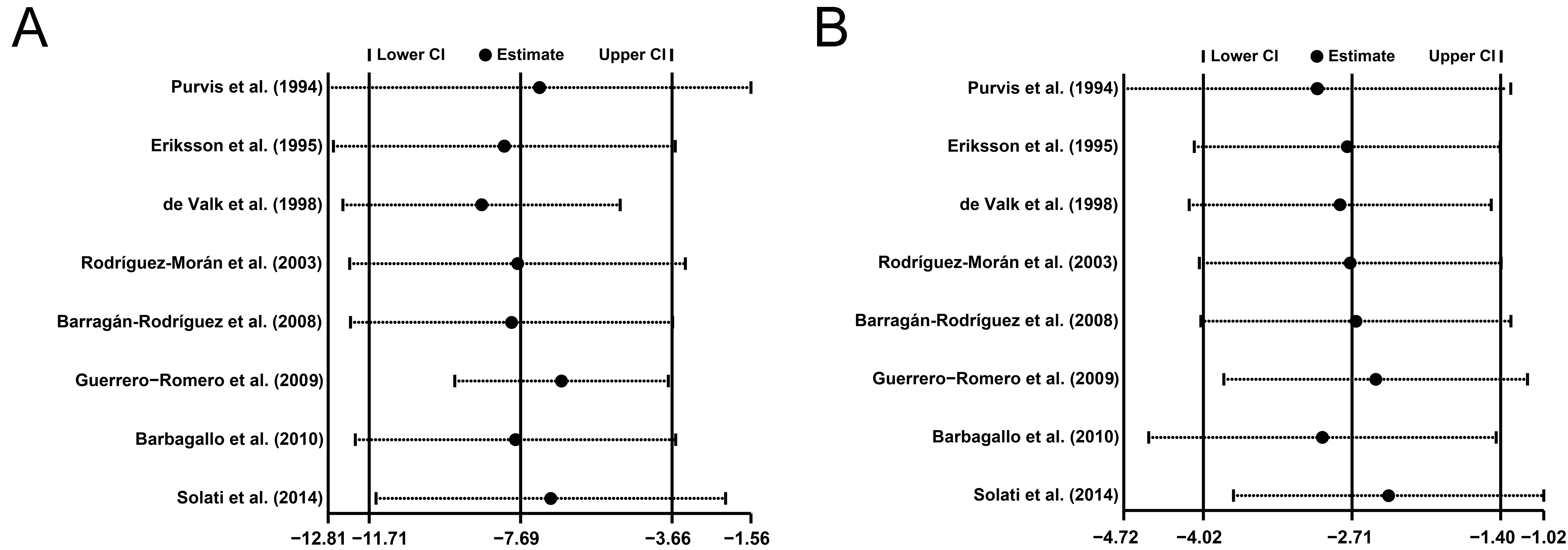
**

**Supplemental Figure 6.** Sensitivity analysis for the included studies about the effects of magnesium supplementation on SBP (A) and DBP (B) in type 2 diabetes mellitus patients. DBP, diastolic blood pressure; SBP, systolic blood pressure.

**2 Supplemental Table**

**Supplemental Table 1.** Quality assessment of the randomized controlled trials included in the meta-analysis.

| Article (first author, year) | Randomized? | Double-Blind? | Withdrawals and drop outs reported? | Randomization method described and appropriate? | Blinding method described and appropriate? | Total Jadad score |
| --- | --- | --- | --- | --- | --- | --- |
| Paolisso et al. (1989a) | 1 | 0 | 1 | 1 | 0 | 3 |
| Paolisso et al. (1989b) | 1 | 1 | 1 | 1 | 0 | 4 |
| Corica et al. (1994) | 0 | 1 | 1 | 0 | 1 | 3 |
| Gullestad et al. (1994) | 1 | 1 | 1 | 0 | 1 | 4 |
| Paolisso et al. (1994) | 1 | 1 | 1 | 1 | 0 | 4 |
| Purvis et al. (1994) | 1 | 1 | 1 | 1 | 1 | 5 |
| Eibl et al. (1995) | 1 | 1 | 1 | 0 | 1 | 4 |
| Eriksson et al. (1995) | 1 | 1 | 1 | 0 | 1 | 4 |
| de Lordes Lima et al. (1998) | 1 | 1 | 1 | 1 | 1 | 5 |
| de Valk et al. (1998) | 1 | 1 | 1 | 0 | 0 | 3 |
| Rodríguez-Morán et al. (2003) | 1 | 1 | 1 | 1 | 1 | 5 |
| Barragán-Rodríguez et al. (2008) | 1 | 0 | 1 | 1 | 0 | 3 |
| Guerrero-Romero et al. (2009) | 1 | 1 | 1 | 1 | 1 | 5 |
| Barbagallo et al. (2010) | 1 | 0 | 1 | 1 | 0 | 3 |
| Bhardwaj et al. (2013) | 1 | 0 | 1 | 1 | 0 | 3 |
| Navarrete-Cortes et al. (2014) | 1 | 1 | 1 | 1 | 1 | 5 |
| Solati et al. (2014) | 1 | 1 | 1 | 1 | 1 | 5 |
| Singh et al. (2015) | 1 | 0 | 1 | 1 | 0 | 3 |
| ELDerawi et al. (2018) | 1 | 0 | 1 | 1 | 0 | 3 |
| Razzaghi et al. (2018) | 1 | 1 | 1 | 1 | 1 | 5 |
| Rashvand et al. (2019) | 1 | 1 | 1 | 1 | 1 | 5 |
| Talari et al. (2019) | 1 | 1 | 1 | 1 | 1 | 5 |
| Rashvand et al. (2020) | 1 | 1 | 1 | 1 | 1 | 5 |
| Sadeghian et al. (2020) | 1 | 1 | 1 | 1 | 1 | 5 |
